# A batch microfabrication of a self-cleaning, ultradurable electrochemical sensor employing a BDD film for the online monitoring of free chlorine in tap water

**DOI:** 10.1038/s41378-022-00359-1

**Published:** 2022-04-08

**Authors:** Jiawen Yin, Wanlei Gao, Weijian Yu, Yihua Guan, Zhenyu Wang, Qinghui Jin

**Affiliations:** 1 Faculty of Electrical Engineering and Computer Science, Ningbo University, 315211 Ningbo, P. R. China; 2 State Key Laboratory of Transducer Technology, Shanghai Institute of Microsystem and Information Technology, Chinese Academy of Sciences, 200050 Shanghai, P. R. China

**Keywords:** Electrical and electronic engineering, Chemistry

## Abstract

Free chlorine is one of the key water quality parameters in tap water. However, a free chlorine sensor with the characteristics of batch processing, durability, antibiofouling/antiorganic passivation and in situ monitoring of free chlorine in tap water continues to be a challenging issue. In this paper, a novel silicon-based electrochemical sensor for free chlorine that can self-clean and be mass produced via microfabrication technique/MEMS (Micro-Electro-Mechanical System) is proposed. A liquid-conjugated Ag/AgCl reference electrode is fabricated, and electrochemically stable BDD/Pt is employed as the working/counter electrode to verify the effectiveness of the as-fabricated sensor for free chlorine detection. The sensor demonstrates an acceptable limit of detection (0.056 mg/L) and desirable linearity (*R*^2^ = 0.998). Particularly, at a potential of +2.5 V, hydroxyl radicals are generated on the BBD electrode by electrolyzing water, which then remove the organic matter attached to the surface of the sensor though an electrochemical digestion process. The performance of the fouled sensor recovers from 50.2 to 94.1% compared with the initial state after self-cleaning for 30 min. In addition, by employing the MEMS technique, favorable response consistency and high reproducibility (RSD < 4.05%) are observed, offering the opportunity to mass produce the proposed sensor in the future. A desirable linear dependency between the pH, temperature, and flow rate and the detection of free chlorine is observed, ensuring the accuracy of the sensor with any hydrologic parameter. The interesting sensing and self-cleaning behavior of the as-proposed sensor indicate that this study of the mass production of free chlorine sensors by MEMS is successful in developing a competitive device for the online monitoring of free chlorine in tap water.

## Introduction

The safety of drinking/tap water is closely related to human health because many toxic and harmful substances, such as viruses, bacteria, heavy metals, microorganisms, and chlorine, can be spread in tap water and endanger personal safety,^1–3^. In China, the content of free chlorine, which is used to kill bacteria and viruses in drinking water, is considered one of the most important indicators to evaluate the safety of tap water. Therefore, chlorine is the main additive in tap water treatment. In reality, an excess addition of chlorine during water treatment can produce carcinogenic substances, such as chloroform, that endanger human health after ingestion^4^. An insufficient addition of chlorine will cause the proliferation of bacteria and microorganisms^5^. Finding the appropriate addition of chlorine to water is always challenging due to the complexity of underground water transportation networks. Thus, there is an urgent need to develop a reliable sensing device to monitor the content of free chlorine in water at each node of a water pipe.

For the purpose of monitoring the changes in the free chlorine level in tap water (in situ), these sensing devices should not only be sensitive to free chlorine but also have reliable performance, nontoxicity, and satisfactory integration. In addition, the distribution of a sensor matrix or network is generally set in a water pipe system or water source so that comprehensive information on water quality can be conveniently obtained; these systems require sensors to have satisfactory long-term stability and some ability for self-cleaning in complex water compositions. Thus, many methods/sensors for free chlorine detection, such as laboratory contaminant analysis and colorimetric contaminant detection, have difficulty meeting the demands of gridding and distributed free chlorine monitoring^6,7^. Electrochemical sensors have many advantages, such as their smaller size and fast response time; thus, their use is considered a possible strategy for the online monitoring of tap water. Milica Jović et al. presented a low-cost, reliable and sensitive electrochemical method for free chlorine analysis in water using inkjet-printed silver electrodes that demonstrated a range of 1–100 mg/L and a limit of detection (LOD) of 0.4 mg/L^8^. Fumihiro Kodera et al. reported an electrochemical sensor for free chlorine detection using nickel-metal nanoparticles combined with a multilayered graphene nanoshell as the sensing electrode^9^. The strategy mentioned above used toxic metals (Ag and Ni) as sensitive electrodes to directly contact drinking water; therefore, it could not be used in large quantities for in situ detection of free chlorine in tap water. Ag^+^ and Ni^+^ precipitate during sensing, and excessive amounts of Ag^+^/Ni^+^ (>0.05 mg/L) in drinking water leads to poisoning due to protein inactivation. Thus, some secure/nontoxic sensors/electrodes have been reported. Fumihiro Kodera et al. developed a new analysis method for high concentrations of free chlorine using linear sweep voltammetry with a platinum (Pt) disk electrode, and a good linear relationship in the concentration range of 7.45–89.40 mg/L was obtained^10^. Arif Ul et al. reported a thin gold film-based reusable and reagentless free chlorine sensor with a high sensitivity of 0.327 μA/mgL^−1^ and a linear range of 0–6 mg/L^11^. In addition, boron-doped diamond (BDD) electrodes have been reported frequently because of their low background current, low scale deposition and chemical inertness^12–14^. Michio Murata et al. designed effective BDD electrodes to quantitatively determine free chlorine based on the oxidation current and studied the reduction behavior of free chlorine on a BDD electrode surface^13,14^. Discrete BDD WEs for analyte detection have been reported frequently, but miniaturized, highly integrated and batch microfabricated sensors based on BDD films are rarely reported. In addition, a few studies on the integration of electrochemical sensors on paper or Si substrates for detecting free chlorine have been presented in recent years^15,16^. However, a highly integrated electrochemical sensor for free chlorine along with a self-cleaning ability has rarely been reported. Hence, to monitor the long-term changes in the free chlorine level of tap water (in situ) online, it is necessary to propose an alternative strategy that addresses these concerns.

Herein, we report a highly integrated silicon-glass double-layered electrochemical sensor chip for the determination of free chlorine in tap water. This study provides a corresponding batch preparation method for sensors based on a microelectromechanical system (MEMS). First, a solid-state Ag/AgCl reference electrode (RE) is placed in a prepackaged saturated KCl solution min-tank (liquid-conjugated Ag/AgCl RE) so that a stable working environment for the Ag/AgCl electrode can be provided to ensure a constant reference potential. Second, BDD is relatively electrochemically stable, nontoxic and has a lower background current than other electrodes (such as silver and nickel). Thus, a BDD thin film is fabricated as the working electrode (WE) that directly contacts tap water. It should be emphasized that the BBD electrode produces hydroxyl radicals (·OH) at a fixed voltage to realize electrode self-cleaning by removing organic matter and microorganisms attached to the surface of the electrode^17^. Third, a Pt electrode is chosen as the counter electrode (CE) because of its relative chemical stability. In addition, all these units are integrated on a mini-chip (with an area of <0.3 cm^2^) to decrease the sensor size. Consequently, the sensing behavior of the as-designed sensor is systematically studied in solutions containing different amounts of free chlorine to verify the effectiveness and feasibility of these batch-fabricated electrochemical sensors. In addition, the mechanism behind the generation of hydroxyl radicals (·OH) and self-cleaning of the sensor surface is a particular focus that is discussed in this study.

## Experimental method

### Sensor design

**Fig. 1:**
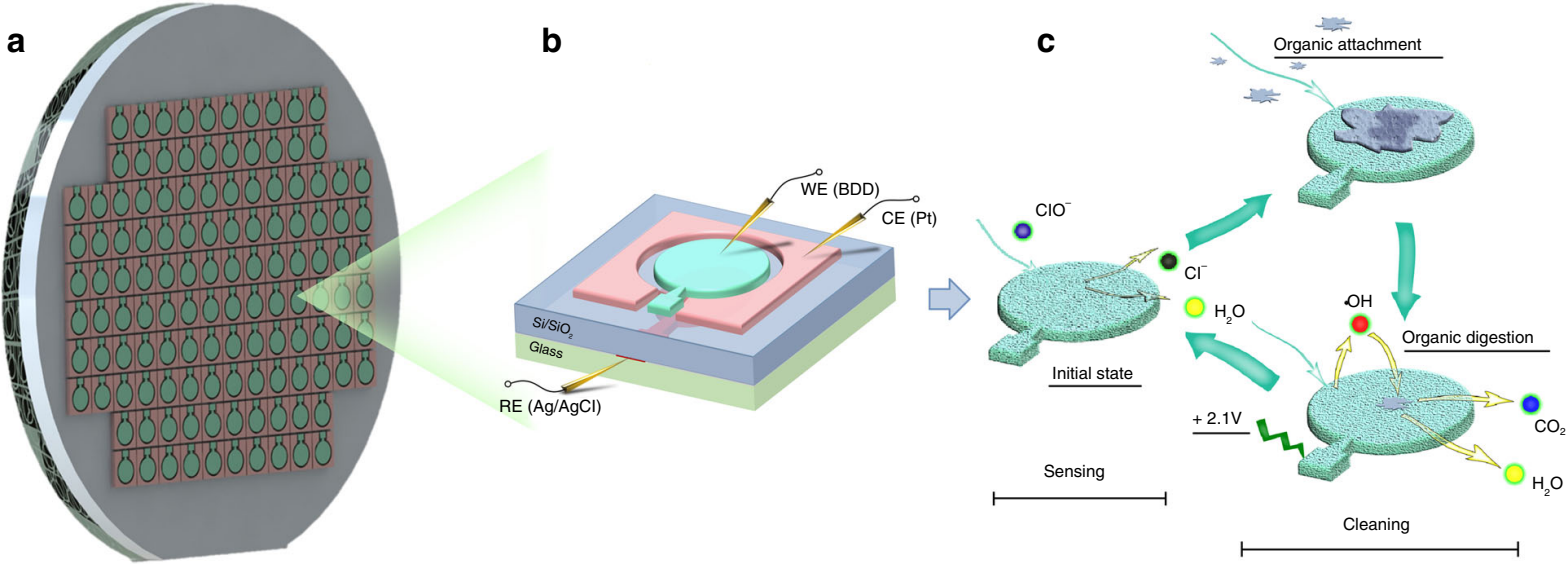
Schematic diagram showing the sensing/cleaning process of the batch microfabricated electrochemical free chlorine sensor. **a** Batch microfabrication using a 4 inch wafer. **b** Diagram of the Si-glass structure of the sensor. **c** Sensing, fouling and self-cleaning process of the sensor

In our previous report, the batch manufactured Ag/AgCl RE with a silicon-glass structure had a more stable reference potential than others because a chloride ion-exchange nanochannel array was designed and fabricated^18^. Consequently, in this paper, a silicon glass-structured, three electrode-integrated electrochemical sensor chip for detecting free chlorine was fabricated by MEMS on a 4 inch wafer. A two-level silicon glass structure was adopted and fabricated, in which a solid-state Ag/AgCl electrode was immersed in saturated KCl solution. In addition, a BDD/Pt thin film was selected as the WE/CE, which was fabricated on the upper surface of silicon (as shown in Fig. 1a). To eliminate/weaken the polarization on the WE and decrease power consumption during the sensing process, the circular WE (the diameter is 4 mm) was surrounded by the CE with a spacing of 300 μm, as shown in Fig. 1b. It is an unavoidable problem that a large amount of organic matter and other substances will adhere to the surface of the sensor and cause a decline in its sensing characteristics. This is a difficult problem to overcome due to the passivation of traditional sensors used as Wes in long-term online monitoring; thus, the development of an ultra-durable free chlorine sensor with a self-cleaning strategy is proposed. Hydroxyl radicals (·OH), which are generated at a fixed voltage on the BDD electrode, can decompose organic matter and microorganisms attached to the electrode surface into carbon dioxide and water (Fig. 1c).

### Sensor fabrication

#### Boron-doped diamond film preparation

A 4 inch oxidized silicon wafer was used as the substrate after ultrasonic cleaning with 75% alcohol for 10 min. A silicon wafer was ground with diamond grinding paste (W28) to produce a large number of disordered, uniform and dense microdefects on the surface. After cleaning with deionized water, the wafer was placed into an acetone suspension of diamond nanopowder (with a diameter of 60–100 nm, from Shanghai Aladdin Biochemical Technology Co., Ltd.) at a concentration of 0.5 g/100 mL and ultrasonicated for 30 min for secondary grinding. In addition, some small nanodiamond particles were embedded in the scratches/pores of the substrate to provide seed crystals for the growth of the diamond film. The seed crystal on the substrate improved the nucleation density of diamond during hot-filament chemical vapor deposition (HFCVD). The preparation of diamond films with a low grain size and low surface roughness became possible after the above two steps. The BDD film electrode was prepared by HFCVD on a resistive silica wafer substrate using a hot-filament temperature of 2200–2400 °C, a reaction pressure of 1.0–3.5 kPa, a carbon/hydrogen ratio of 2–4% and a deposition time of 4.5 h. The boron/carbon source was a mixture of B_2_O_3_ and C_2_H_5_OH and CH_4_.

#### Batch fabrication of the sensor chips on a 4 inch wafer

**Fig. 2:**
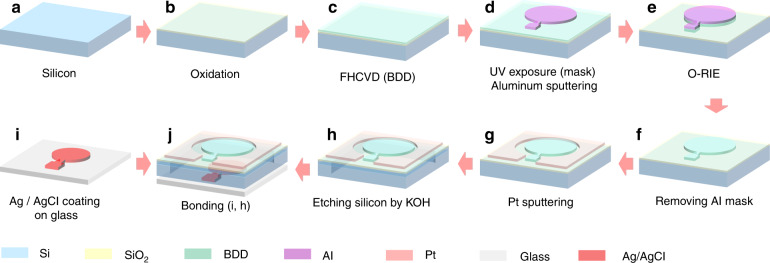
Fabricating process for the as-proposed electrochemical free chlorine sensor. **a** A clean silicon wafer. **b** oxidation process. **c** Preparation of BDD film by FHCVD. **d** Preparation of aluminum mask by a lift-off process. **e** Preparation of BDD electrode by O-RIE process. **f** Removing Al mask. **g** Preparation of Pt electrode by sputtering process. **h** Wet etching of silicon. **i** Preparation of Ag/AgCl electrode. **j** Bonding silicon wafer and glass wafer

Initially, the silicon wafer was thoroughly cleaned by washing with H_2_SO_4_:H_2_O_2_ = 7:1 (Step-1), NH_4_OH:H_2_O_2_:H_2_O = 1:1:7 (Step-2), and HF:H_2_O = 1:50 (Step-3) (Fig. 2a). After that, a (100) crystal-oriented silicon wafer (polished on both sides) with a diameter of 4 inches, a SiO_2_ thickness of ~2 μm was obtained by dry oxidation for 55 min and wet oxidation for 450 min at 1100 °C (Fig. 2b). The silicon wafer was thoroughly cleaned and dried, and then a 3–4 µm thick BDD film was fabricated by HFCVD; the details of this preparation were provided in the previous section (Fig. 2c). Then, a 2.4 μm thick LC100A photoresist from Rohm and Haas was coated with an EVG101 spin coater from Beijing Yake Chenhui Technology Co., Ltd. for 30 s at 1000 r/min. For patterning, the Si/BDD wafer was exposed to an MA6 lithography machine from Germany SUSS Micro Tec for 15 s and immersed in FHD-320 solution from Micro Resist Technology for 40 s. Then, a patterned aluminum (Al) film with a thickness of ~400 nm was obtained by sputtering Al on the Si/BDD wafer followed by a lift-off process with an MSp-3300 instrument from Beijing Chuangshi Weina Technology Co., Ltd. (Fig. 2d). As shown in Fig. 2e, a BDD film electrode under an aluminum mask was obtained after the wafer was placed in oxygen reactive ion etching (O-RIE) equipment (Zepto from Yamato) for 40 min. Afterward, the above wafer was immersed in 80% phosphoric acid at a temperature of 60 °C for 600 s to remove the aluminum mask and expose the BDD electrode (Fig. 2f). A Pt electrode with a thickness of ~0.4 μm was obtained by sputtering Pt on the wafer followed by a lift-off process with the MSp-3300 instrument from Beijing Chuangshi Weina Technology Co., Ltd. (Fig. 2g). At this point, a WE and CE pair was prepared. After repeating the photoresist coating and UV exposure on the back side of the silicon/BDD/Pt wafer, a buffer oxide etching (BOE, HF: H_2_O = 1:50) solution was used to completely etch the SiO_2_ layer for 720 s without protecting the LC100A photoresist. Next, the wafer was etched/to prepare a cavity and groove for placing the 3 M KCl solution and lead wire after immersing it in 30% KOH solution for ~20 h at a temperature of 40 °C due to the different etching rates of Si and SiO_2_ (10.3 μm/h and 0.055 μm/h, respectively), as shown in Fig. 2h.

A 4 inch Pyrex 7740 glass substrate was cleaned by using a standard washing process along with the abovementioned cleaning method used for the silicon wafer and O_2_ plasma treatment. A silver electrode with a thickness of ~0.4 μm was obtained by sputtering Ag on the glass followed by a lift-off process. A certain thickness of AgCl was deposited on the surface of the Ag layer by electrochemical treatment with a 0.25 M HCl solution at a constant voltage of 4 V (Fig. 2i).

Finally, the as-prepared silicon wafer and as-fabricated glass substrate were cleaned for 30 min using a 900 kHz megasonic MC01 instrument from the German Frist-Nano System Limited. After precise alignment, the silicon glass was bonded by SB6e from Germany SUSS Micro Tec (35 min, 350 °C, and 1200 V) to form the final sensor chip (Fig. 2j).

### Evaluating the sensing behavior

The sensing behavior of the microfabricated sensor was evaluated by the following aspects. Initially, the sensor was operated in cyclic voltammetry (CV) mode at a voltage scan rate of 100 mV/s and free chlorine concentrations of 5–200 mg/L to investigate the sensing behavior of free chlorine on the as-fabricated BDD electrode surface. After that, a flow injection experiment was set up to analyze the content of free chlorine at a lower concentration (from 0.1 mg/L to 1 mg/L). In addition, the relationships among the pH, temperature, water flow rates and analyte solution were investigated. Considering that the sensors were fabricated by the MEMS technique, the consistency of the sensors was explored. Third and foremost, the optimal BDD electrode potential for generating hydroxyl radicals is investigated. The self-cleaning process of the sensor was observed dynamically by electrochemically detecting free chlorine at a constant concentration of 100 mg/L.

## Results and discussion

### Characterization of the BDD electrode by SEM and AFM

**Fig. 3:**
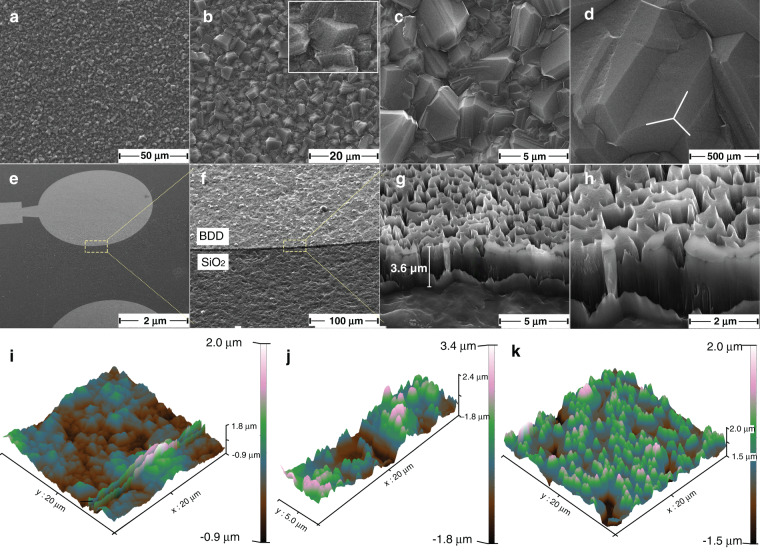
SEM of the as-fabricated BDD film/electrode of the free chlorine sensor. **a**–**d** Surface morphology of the BDD film fabricated by FHCVD at 2000× (**a**), 5000× (**b**), 20,000× (**c**), and 160,000× (**d**). **e**–**h** Surface morphology of the BDD electrode after O-RIE. **i** AFM image of the BDD film surface before O-RIE. **j** AFM image of the BDD electrode boundary. **k** AFM image of the BDD film surface after O-RIE

In this work, BDD was selected as the WE of the sensor and fabricated by FHCVD. Scanning electron microscopy (SEM) and atomic force microscopy (AFM) images of the as-fabricated BDD film/electrode were taken to characterize the BDD (Fig. 3). We can clearly see that the BDD film is uniformly prepared on the surface of SiO_2_ with a typical octahedral diamond morphology in which many triangle/square crystal faces are observed (insert of Fig. 3b and i). This result is highly consistent with other studies when CH_4_ is used as a carbon source^19,20^. In addition, a desirable square resistance (0.5 Ω) is found by the ST2558A-F02 instrument from Suzhou Jingge Electronic Co., Ltd. (BDD thickness of 3.5 µm). Therefore, the fabricated BDD film demonstrates acceptable resistivity (1.8 e^−5^ Ω/cm). The diamond in the non-WE area is removed through the O-RIE process using aluminum film as a mask (diameter of 3 mm and thickness of 400 nm). Figure 3e–h shows the surface morphology of the BDD electrode after O-RIE. In the brighter region, the BDD is preserved because it is protected by the aluminum mask. In the darker region, the BDD is etched by the high-speed, directional movement of oxygen ions, thus exposing the SiO_2_ substrate (Fig. 3e, f, and j). A high-resolution photo of the BDD electrode was obtained, as shown in Fig. 3g and h. However, an unexpected phenomenon is found in which the surface of the BDD changes from a convex cone polyhedron to a concave gully (comparing Fig. 3c with Fig. 3g), which is similar to the study of Gopi M.R. et al.^21^. It is considered that the oxygen ions moving at high speed produce a higher temperature (>660 °C), which leads to the melting of aluminum on the surface of the BDD. With the continuous thinning of the aluminum mask, the sharp cone-shaped diamond particles lose their aluminum protection (melted) and are consumed by the oxygen ions, similar to cutting a mountain peak, as shown in Fig. 3g and h. Comparing Fig. 3i and Fig. 3k, the surface area and R_a_ (roughness) of the BDD increase by 1.65 times (556 μm^2^ vs. 920 μm^2^) and 2.08 times (175 nm vs. 365 nm) after O-RIE, respectively. This is exciting as it results in better sensing characteristics.

### Sensing behavior of the as-proposed free chlorine sensor

**Fig. 4:**
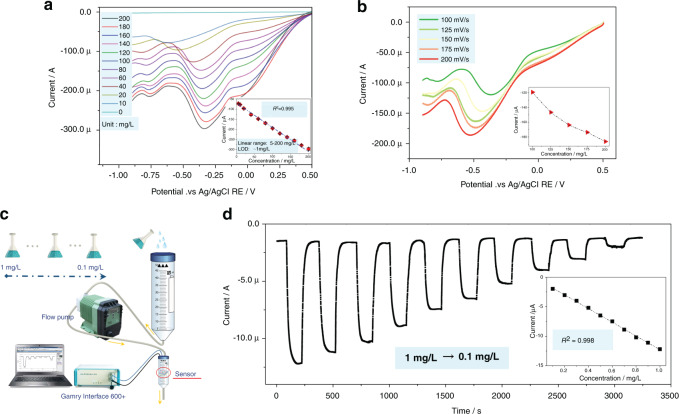
Sensing characteristics of the as-fabricated free chlorine sensor. **a** Sensing characteristics of the proposed sensor operated in CV mode with a voltage sweep rate of 100 mV/s. Insets are the dependence of the response signal on NaClO concentration. **b** Influence of the scan rate on the response signal of the proposed sensor. **c** Schematic diagram of the experimental flow injection device. **d** Sensing behavior of the fabricated sensor operated with the amperometric method. Insets are the dependence of the response signal on the free chlorine concentration

Silicon-based Res have demonstrated desirable performance in previous studies, and they have been proven to provide a stable reference potential for electrochemical detection^18,22^. Therefore, performance testing of the RE for free chlorine will not be conducted in this paper. Initially, the sensor was operated in CV mode at a scan rate of 100 mV/s to study the impact of the applied bias voltage and dependency of the response signal on the free chlorine concentration, as shown in Fig. 4a. With the bias voltage applied on the BDD WE ranging from 0.5 to −0.9 V and −0.9 to 0.5 V, a maximum response value is observed at a bias voltage of ~−0.35 V. ClO^−^ is reduced near the BDD surface^13,14^, as indicated below:

1 $${\rm{ClO}}^- + {\rm{H}}_{2}\rm{O} +2{\rm{e}}^-\to 2{\rm{OH}}^-+{\rm{Cl}}^{-}$$

Accordingly, the electrochemical reaction shown in Eq. (1) is triggered at a bias voltage of ~−0.35 V and most of ClO^−^ is reduced at this potential. In addition, the dependence of the response signal on the NaClO concentration is considered and shown in the inset of Fig. 4a. The equation for the line fitted by the ClO^−^ concentration and the electric current peak value (*I*) can be interpreted as [Eq. 2]:

2 $$I = - \left( {1.144 \times \alpha \left( {ClO^ - } \right) + 71.193} \right)$$

where *I* is the value of the electric current peak (μA), and α(ClO^−^) is the concentration of free chlorine. Satisfactory linearity (*R*^2^ = 0.995) is observed in the NaClO content range of 5–200 mg/L, implying a desirable sensing accuracy. Interestingly, the sensitivity of the BDD film for free chlorine sensing in this work is higher than that in other studies perhaps because of the large specific surface area of the BDD electrode (this work: 9.108 μA/mgL^−1^/cm^2^, Ref. ^14^:0.744 μA/mgL^−1^/cm^2^, Ref. ^13^:1.912 μA/mgL^−1^/cm^2^).

It can be confirmed that a quick scan rate indeed enhances the response value (Fig. 4b); for instance, the maximum response value to 80 mg/L NaClO obtained at a scan rate of 200 mV/s (~186 μA) is almost 1.6 times higher than that of the value obtained at a scan rate of 100 mV/s (~119 μA). However, an excessively quick scan rate results in the maximum response value appearing at a higher bias voltage. Since an ultrahigh bias voltage will decrease the stability of the sensor, a scan rate of 100 mV/s is selected to maintain the high response value and stability of the sensor.

Generally, the concentration of free chlorine in the tap water of China is 0.05–0.8 mg/L. Unfortunately, the LOD of the as-fabricated sensor is ~1 mg/L in CV mode. Consequently, a flow injection device was designed to realize free chlorine sensing at a lower concentration. As shown in Fig. 4c, two centrifugal tubes (50 mL and 5 mL) were connected to a flow pump with hose. The sensor was placed in a 5 mL centrifuge tube and sealed with glue.

A potential of −0.35 V vs. Ag/AgCl RE, which was determined from Fig. 4a, was fixed for the flow injection analysis (FIA) with a solution of 0.01 M PBS at pH 8.5. Initially, with the continuous injection of deionized water, a relatively stable current value of ~1.5 μA is obtained from 0 to 85 s. Then, an increase in the current value by 10.9 μA is observed after injecting 1 mg/L NaClO solution into the sensor at a fixed flow rate of 20 mL/min (90–120 s). After a stabilization period of ~80 s (120–200 s), a significant drop in the current value is observed when injecting deionized water into the device again. Finally, NaClO solutions with concentrations of 0.9 mg/L, 0.8 mg/L, 0.7 mg/L, 0.6 mg/L, 0.5 mg/L, 0.4 mg/L, 0.3 mg/L, 0.2 mg/L, and 0.1 mg/L were injected into the FIA device before and after the injection of deionized water (205–3200 s), and the fluctuation in current is shown in Fig. 4d. The equation of the line fitted by the ClO^−^ concentration and electric current value can be shown as [Eq. 3]:

3 $$I = - \left( {11.602 \times \alpha \left( {ClO^ - } \right) + 0.682} \right)$$

A linear dependency (*R*^2^ = 0.998) of the free chlorine concentration on the response signal is shown in the inset of Fig. 4d. In addition, an LOD of 0.056 mg/L free chlorine was calculated based on the rule of a signal-to-noise ratio of 3. This means that the performance of the as-proposed sensor can meet the requirements of detecting/monitoring free chlorine in tap water.

### Effects of the pH, flow rate and temperature on the sensing behavior of the prepared sensor

**Fig. 5:**
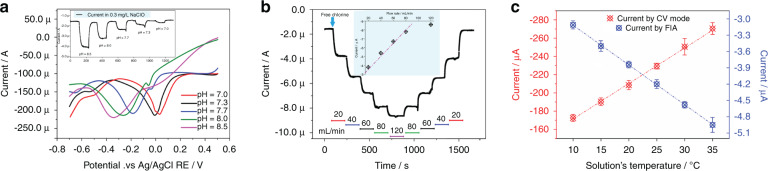
Effects of flow rate, temperature and pH on the sensing characteristics of the sensor. **a** Response signal in 100 mg/L NaClO at different pH (7.0–8.5) with a voltage sweep rate of 100 mV/s in CV mode. Insets are the response current in 0.3 mg/L NaClO at different pH (7.0–8.5) by FIA. **b** Response signal in 0.3 mg/L NaClO at different flow rates (20–120 mL/min) and a pH of 8.5 by FIA. Insets are the dependency of the flow rate and response signal. **c** Dependency of the temperature and response signal in 180 mg/L NaClO using CV mode and in 0.3 mg/L NaClO using FIA, respectively, at a pH of 8.5 and flow rate of 20 mL/min (*n* = 3)

Generally, the pH of tap water fluctuates between 6.8 and 8 in China^23^. In this paper, an electrochemical sensor for in situ/online detection of free chlorine in tap water is reported. The factors affecting free chlorine sensing in tap water must be comprehensively investigated to ensure accurate sensing. Electrochemical-based chlorine detection is influenced by the solution pH, which defines the particular ionic species of chlorine present in solution. Hence, an experiment was set up to verify the degree of influence of pH on the detection of free chlorine by the as-proposed sensor. A series of PBS buffers at different pH values (from 7.0 to 8.5) were prepared by adjusting the ratio of NaH_2_PO_4_ to Na_2_HPO_4_. The dependence of the response behavior on pH is tested with 100 mg/L free chlorine by CV and 0.3 mg/L free chlorine by FIA; the results are shown in Fig. 5a and the inset of Fig. 5a, respectively. Figure 5a shows that the reduction peak of the CV curve obviously shifts to the right (from −360 mV to 30.1 mV) with decreasing alkalinity (from 8.5 to 7.0), while the electric current value of the reduction peak remains constant (−215.6 μA ± 4.5 μA). In FIA mode, the response current at different pH values (7.0–8.5) with a NaClO concentration of 0.3 mg/L and a constant potential of −0.35 V is shown in the inset of Fig. 5a, and the attenuation of the response signal is observed with decreasing pH. The optimal reaction potential is not −0.35 V in a low pH solution due to the right shift of the CV peak. The dependency of the response signal on the pH conforms to Eq. (4) in an alkaline solution atmosphere:

4 $$I = 0.5 \times pH^2 - 6.11 \times pH + 19.71$$

Fortunately, the relationship between pH and the response signal in FIA mode is linear (*R*^2^ = 0.988), so the deviation of the response current caused by pH fluctuation can be compensated by Eq. (4) in online applications.

Another factor affecting the sensing of free chlorine is the flow rate of tap water. Then, an FIA experiment was established to investigate the effect of the flow rate on free chlorine sensing through the accurate control of the solution flow rate by a pump with a constant voltage of −0.35 V. As shown in Fig. 5b, an increase in the current value by ~2.2 μA is observed after the injection of 0.3 mg/L NaClO solution into the sensor (100–105 s) at a flow rate of 20 mL/min and a pH of 8.5. After a stabilization period of ~50 s (110–250 s), an increase in the current value by ~1.6 μA is observed when changing the flow rate from 20 mL/min to 40 mL/min. After that, the current value increases (1.3 μA, 1.1 μA, and 0.9 μA) continuously with increasing flow rate (60 mL/min, 80 mL/min, and 120 mL/min). The equation for the line fitted between the flow rate (*V*) and electric current value (*I*) in 0.3 mg/L NaClO can be interpreted as [Eq. 5]:

5 $$I = - 0.067 \times V-2.6$$

We conjecture that the enhanced response with increasing flow rate may be caused by more ClO reaching the surface of the BDD electrode during sensing. However, we only fit the dependence at a lower flow rate because the free chlorine flows away too quickly at a higher flow rate; thus, the reaction does not take place. The satisfactory linearity (*R*^2^ = 0.983) means that the influence of the flow rate on free chlorine detection can be predicted and eliminated in the future.

The temperature change of tap water can exceed 20 °C (10–30 °C) within 1 year. Therefore, the influence of temperature on the free chlorine sensor must also be considered. The dependency of the temperature and response signal in 100 mg/L NaClO using CV mode and in 0.3 mg/L NaClO using FIA is shown in Fig. 5c. An obvious enhancement in the response signal for free chlorine detection is observed at a higher solution temperature regardless of which mode (CV/FIA) is used. An ~19.5 μA increase in the response current is found after the solution temperature is increased by 5 °C in 100 mg/L NaClO, and the equation for the line fitted between (*R*^2^ = 0.998) temperature (*T*) and current (*I*) can be interpreted as [Eq. 6]:

6 $$I = - \left( {3.89 \times T + 132.4} \right)$$

Similar to the above, the dependency (*R*^2^ = 0.995) of the temperature and response signal conforms to Eq. (7) in 0.3 mg/L NaClO when using FIA mode:

7 $$I = - \left( {0.073 \times T + 2.384} \right)$$

Such satisfactory linearity indicates the excellent characteristics of the as-proposed free chlorine sensor.

### Consistency, reproducibility and long-term stability of the as-proposed sensor

**Fig. 6:**
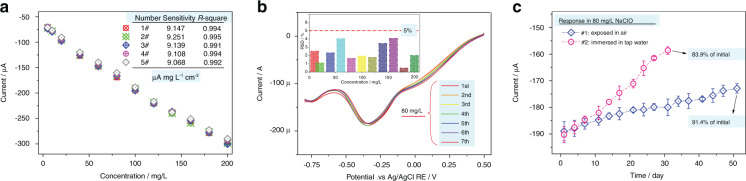
Consistency, repeatability and working life of sensors. **a** Response signal for each sensor obtained via the microfabricated technique to 10–200 mg/L NaClO in CV mode; **b** CV curves showing the response to 80 mg/L NaClO in parallel tests using five sensors. Insets are the RSD of the peak current for each concentration of NaClO solution (*n* = 7). **c** Stability test of the sensor (50 days)

Since the sensor is mass-produced via a micro/nanomanufacturing technique, the response consistency for those sensors is a concerning issue. Herein, five sensors fabricated with the same microfabrication technique were selected as representative examples, and their response behavior were compared. As shown in Fig. 6a, the sensitivity of the five sensors for free chlorine detection in CV mode is 9.147 μA/mgL^−1^/cm^−2^, 9.251 μA/mgL^−1^/cm^−2^, 9.139 μA/mgL^−1^/cm^−2^, 9.108 μA/mgL^−1^/cm^−2^, and 9.068 μA/mgL^−1^/cm^−2^. The linearity of the fitted line between the ClO^−^ concentration and peak current of the five sensors is >0.99 from 10 mg/L to 200 mg/L. The extremely consistent sensitivity and R-square of the as-proposed sensors indicate that the fabrication of a free chlorine sensor by MEMS is successful.

The reproducibility of the response value of the proposed sensor to free chlorine from 5 to 200 mg/L can be seen in Fig. 6b and the insets. Small RSDs (<4.05%) are found for the sensor operated under the same measurement conditions (*n* = 7).

A desirable long-term stability is the primary characteristic of a sensor working online. Hence, for the purpose of verifying the durability of the sensor, a continuous 50-day parallel experiment was designed and implemented (Fig. 6c). After 50 days of continuous measurement, the sensor exposed to air in the no-working period retains a 91.4% response value (−189.2 μA vs. −172.9 μA) compared with that of the sensor at its initial stage. This implies a great improvement in the period of validity for future microfabricated electrochemical-free chlorine sensors. However, the sensor immersed in tap water in the no-working period shows unsatisfactory long-term stability and only retains an 83.9% response value (−190.3 μA vs. −158.6 μA) after 30 days when compared with that at its initial stage. The minor decrease in the response of the sensor when exposed to air is mainly attributed to the adhered organics/microbes on the surface of the BDD WE. Therefore, further activation/cleaning is necessary for the sensor after continuous operation for 1 month. Thus, the proposed sensor features satisfactory stability and desirable response consistency as well as high reproducibility in its sensing behavior, offering the opportunity for mass production and application in the future.

### Investigation on the self-cleaning process of the BDD electrode

Dissolved organic matter (DOM) is considered a heterogeneous matrix of organic contents with different chemical structures, functionalities, and molecular weights. Humic acid (various aromatic compounds) accounts for the vast majority of DOM in tap water and mainly comes from the metabolism of microorganisms^24,25^. This leads to the formation of biofilms on the inner wall of the pipe/sensor surface (as shown in Fig. 1c). The sensing ability of the sensor will deteriorate after biofilm attachment, so it is necessary to develop a sensor with self-cleaning characteristics. BDD electrodes have been widely used in wastewater treatment because of their satisfactory electrochemical digestion potential^26,27^. In view of its electrochemical digestion behavior, BDD was selected and prepared as the sensitive electrode in this work to realize the in situ self-cleaning of the sensor. Therefore, the process of digesting organic matter (self-cleaning) on the surface of BDD is discussed in this section. It has been reported that BDD electrodes can generate hydroxyl radicals (•OH) with high current efficiency during anodic water decomposition^25^ [Eqs. 8, 9]:

8 $${\rm{H}}_2{\rm{O}} \to \bullet {\rm{OH}} + {\rm{H}}^+ +{\rm{e}}^-$$

Hydroxyl radicals are strong oxidants capable of oxidizing organic compounds to carbon dioxide and water:

9 $${\rm{Organic}}\,{\rm{matter}} + \bullet {\rm{OH}} \to {\rm{CO}}_2 + {\rm{H}}_{2}{\rm{O}}+ {\rm{x}}$$

**Fig. 7:**
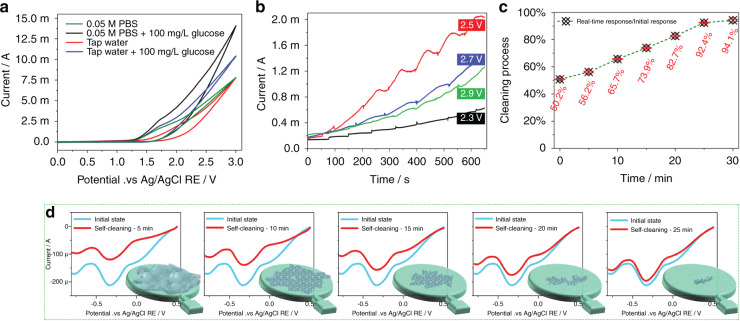
Self cleaning characteristics of as-proposed sensor. **a** CV for 100 mg/L glucose in 0.05 M PBS and tap water at the BDD WE, respectively. **b** Response current to the injection of 200 μL of 100 mg/L glucose samples, at a frequency of ~80 s, in 5 mL of tap water that is stirred at 200 rpm and with different anode voltages. **c** Recovery of the sensing performance of the sensor during sensor self-cleaning. **d** CV curve of the fouled sensors in 100 mg/L NaClO solution after electrochemical cleaning for 5 min,10 min,15 min,20 min, and 25 min at an anode potential of +2.5 V vs. Ag/AgCl RE

Optimization of the electrode potential of the sensor for electrode self-cleaning is the first step. Figure 7a shows the CV curves of 100 mg/L glucose in 0.05 M PBS and tap water, respectively. Figure 7a shows that the onset of the OER current is ~+1.7 V vs. Ag/AgCl, and no obvious anodic current for glucose oxidation is observed at a lower positive potential. Although the current response in tap water is lower than that in PBS buffer due to the lower conductivity, the possibility of the BDD generating •OH in tap water is proven.

Next, we notice a larger current difference at a high positive potential (>+2 V) when comparing the CV curves of the 100 mg/L glucose and bare samples. As shown in Fig. 7b, at a constant electrode potential below 2.3 V with stirring at 200 rpm, no obvious current response is observed after the addition of 200 μL of 100 mg/L glucose solution in 5 mL of tap water at a frequency of ~80 s. A constant electrode potential of 2.5 V, 2.7 V, and 2.9 V is successively observed with the same experimental process as above. The current increase after the addition of glucose represents the •OH generation rate of the BDD electrode. Therefore, we ultimately decide on an optimal electrode potential of 2.5 V for sensor self-cleaning.

River water (Yong River, Ningbo) was filtered and sent to Qingdao Hengli Testing Co., Ltd., to detect the concentration of organic matter. The organic concentration of the measured river water is 14.5 mg/L by the potassium dichromate method. Six sensors were immersed in river water for 2 months to generate organic biofilms on the BDD. These sensors were then fully cleaned with deionized water (no ultrasonication) before the self-cleaning experiment. A significant reduction (almost half) in the sensing capability of the sensor is observed when comparing the response current of the biofilm-attached sensor with the pristine sensor. Therefore, a constant potential of +2.5 V vs. Ag/AgCl RE was applied to the six sensors in tap water for 5, 10, 15, 20, 25, and 30 min to electrochemically clean the BDD electrode. Rice, D. used an OCT microscope to characterize the digestion process of the biofilm on the BDD^28^, and we evaluated the cleaning process of the electrode through the recovery of the sensing behavior for the same analyte in this work. The process of recovering the sensing capability of the sensor during self-cleaning is displayed in Fig. 7c. The response of the sensor is only 50.2% at the initial state (test the response immediately after fabrication) when immersed in river water for 2 months. The details of self-cleaning are shown in Fig. 7d. As cleaning proceeds, the peak current of the sensor in 100 mg/L NaClO solution increases because the biofilm attached to the BDD electrode is digested by •OH. The corresponding relationship between the response signal in 100 mg/L NaClO and the cleaning time is (5 min, −119.3 μA), (10 min, 139.5 μA), (15 min, 156.8 μA), (20 min, 176.9 μA), and (25 min, 197.1 μA); the initial response signal of the sensor is ~212.3 μA. After 30 min, the performance of the sensor recovers to 94.1% of the initial state, which means that the attached biofilm on the sensor surface has been removed. This self-cleaning strategy provides a solution for online free chlorine monitoring of tap water by avoiding sensor damage due to the adhesion of a biofilm or passivation of the electrode. This promising result of BDD electrode self-cleaning offers the opportunity for long-term mass application of free chlorine sensors in situ. In addition, the organic concentration in tap water is lower than that in river water (1–3 mg/L vs. 14.5 mg/L), which means that the cleaning frequency can be appropriately reduced if the sensor is used in tap water. The cleaning frequency of the sensor in tap water will be quantified in future studies.

### In situ/online monitoring of free chlorine in tap water using the as-proposed sensor

**Fig. 8:**
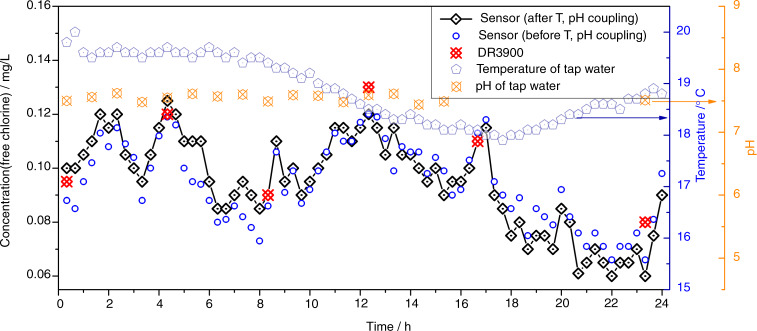
Monitoring the free chlorine concentration of tap water in the pipeline in situ for 24 h

The abovementioned results confirm the high accuracy, acceptable response time, and favorable linearity for detecting free chlorine with the proposed sensor. However, whether these attractive sensing characteristics can be maintained is still a question when compared with other free chlorine detection devices, e.g., DR3900 (spectrophotometer purchased from Hash) and LS5 (spectrophotometer purchased from Yidian Scientific Instrument Co., Ltd.). N,N-diethyl-p-phenylenediamine was used as the chromogenic agent in the spectrophotometry (DR3900 and LS5) tests, and its chromogenic wavelength is 530 nm. Table 1 shows the comparison of the spectrophotometry method and electrochemistry method (this paper) for free chlorine detection. As shown in Table 1, the maximum deviation in free chlorine detection is obtained by comparing with the theoretical value, and DR3900/LS5 is only 0.03 mg/L, displaying satisfactory consistency. In addition, the working state of the sensor in a water pipeline is attractive. The free chlorine sensor is bound to a temperature sensor (PT100, from Shanghai Yidian Scientific Instrument Co., Ltd.) and fixed in a water pipe outside the laboratory. A pH sensor (PHS-25, from Shanghai Yidian Scientific Instrument Co., Ltd.) was used to measure the pH of the tap water offline at a sampling frequency of 60 min. A circuit module was used as the sensor power supply for data collection at a sampling frequency of 20 min. The water flow rate through the water pipe was 0 mL/min during each measurement. The response of the sensors after continuous testing for 24 h (8:00 to 8:00 the next day, Beijing time) is shown in Fig. 8. As shown in Fig. 8, a satisfactory result is witnessed: the content of free chlorine in tap water fluctuates at ~0.1 mg/L (0.06–0.11 mg/L) after coupling with the temperature and pH data through Eqs. (4) and (7) due to the difference in water consumption at the end of the pipe network in different time periods. At 22:00 to 7:00 the next day, the free chlorine level is lower because more free chlorine is consumed by microorganisms, due to the tap water not being released. Fortunately, it is still greater than the minimum value specified in the national standard of tap water in China (GB 5749-2006, >0.05 mg/L). Such interesting results suggest that the proposed sensor has the potential to sense free chlorine in tap water.

**Table 1:** Detecting free chlorine level via spectrophotometer and as-proposed sensor

NaClO	DR3900		LS5		As-fabricated sensor	
mg/L	Absorbance A	Concentration mg/L	Absorbance A	Concentration mg/L	Current μA	Concentration mg/L
0	0.006	0.00	0.004	0.00	−1.13	0.00
0.1	0.045	0.09	0.018	0.10	−1.98	0.09
0.3	0.151	0.30	0.063	0.29	−4.06	0.28
0.5	0.264	0.51	0.107	0.50	−6.55	0.49
0.7	0.372	0.69	0.152	0.70	−8.93	0.70
0.9	0.482	0.89	0.196	0.91	−11.11	0.88

## Conclusion

A silicon glass-structured three electrode-integrated electrochemical sensor chip was designed and fabricated by MEMS, exhibiting batch processing, high precision, antibiofouling/antiorganic passivation and ultradurability for the in situ monitoring of free chlorine in tap water. By fabricating a thin BDD WE on a Si/SiO_2_ substrate and integrating a liquid-conjugated Ag/AgCl RE and Pt CE together, the microfabricated electrochemical sensor showed satisfactory sensing performance for detecting free chlorine, such as a desirable response sensitivity, an acceptable LOD, broad linearity, satisfactory consistency and repeatability, and particularly satisfactory long-term stability. The influence of pH, flow rate and temperature on the sensing behavior of the prepared sensor was explored and showed a linear dependency. Particularly, satisfactory self-cleaning characteristics were observed for the proposed sensor, proving the potential of the sensor for long-term online monitoring. However, the surface topography of the BDD film changed from a convex cone polyhedron to a concave gully after O-RIE. Although the reason for this phenomenon was explained/assumed by the melting of the aluminum mask at high temperature and the sensing characteristics of the as-proposed sensor were satisfactory, it is still worth putting more effort into identifying the generation mechanism of the concave gullies.

## Supplementary information

Abstract Graphical
