# Supplementary figures and images for: A batch microfabrication of a self-cleaning, ultradurable electrochemical sensor employing a BDD film for the online monitoring of free chlorine in tap water
Source: Microsyst Nanoeng. 2022 Apr 8;8:39. doi: 10.1038/s41378-022-00359-1 (PMC8993810; doi:10.1038/s41378-022-00359-1)

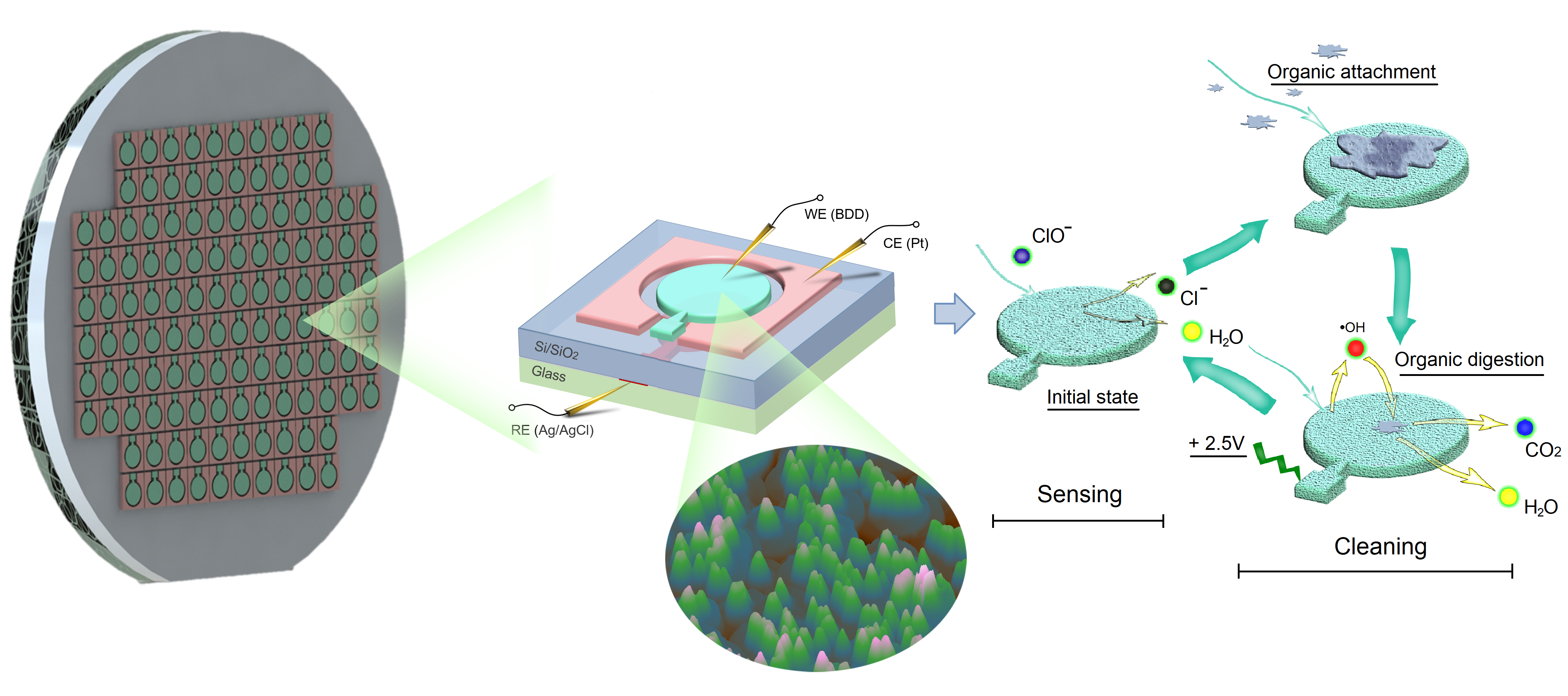

Supplement: Supplementary file 1 — Abstract Graphical [file 41378_2022_359_MOESM1_ESM.tif]
